# Orbital Hematoma Treatment—A Retrospective Study

**DOI:** 10.3390/jcm13195788

**Published:** 2024-09-28

**Authors:** Bartosz Bielecki-Kowalski, Natalia Bielecka-Kowalska, Marek Jaxa-Kwiatkowski, Krzysztof Osmola, Marcin Kozakiewicz

**Affiliations:** 1 Department of Maxillofacial Surgery, Medical University of Lodz, 113 Żeromskiego St., 90-549 Lodz, Poland; bartosz.bielecki-kowalski@umed.lodz.pl (B.B.-K.); marcin.kozakiewicz@umed.lodz.pl (M.K.); 2 Department of Oral Mucosal and Periodontal Diseases, Medical University of Lodz, 251 Pomorska St., 92-213 Lodz, Poland; 3 Clinical Department of Maxillofacial Surgery, Medical University of Poznan, 49 Przybyszewskiego St., 60-355 Poznan, Poland; marek.jaxa.kwiatkowski@gmail.com (M.J.-K.); krzysztof.osmola@usk.poznan.pl (K.O.)

**Keywords:** orbital hematoma, orbital compartment syndrome, optic nerve neuropathy, orbital injury

## Abstract

**Background:** Bleeding within the orbit in the form of a subperiosteal or retrobulbar hematoma is a relatively common complication of trauma and surgery. It affects up to 30% of patients fractures involving the orbital bones. Most cases do not require surgical intervention because they do not cause retinal ischemia or optic nerve neuropathy. The above symptoms occur in only 0.5–1% of patients developing Orbital Compartment Syndrome (OCS). Due to the short period (60–100 min) of time in which the optic nerve and retina can tolerate increased intraocular pressure, it seemed reasonable to evaluate and standardize the surgical management protocol for this rare complication. **Objective:** The aim of this retrospective study was to retrospectively analyze cases of inframammary haematomas with clinically relevant correlations. **Methods:** Eighteen patients treated at the Department of Maxillofacial Surgery due to OCS, in Lodz and Poznan, Poland, between 2009 and 2022, were included. APTT, INR, systemic diseases, cause, location and size of hematoma, presence and number of fractures, visual disturbances and pupillary response on the day after surgery and one month after, the type of intervention and time between admission to the hospital and surgery were evaluated. **Results:** Statistically significant correlations were obtained between the size of the hematoma and the patients’ age, the degree of visual disturbance and the weakening of pupillary constriction, severe initial symptoms and poor postoperative outcomes at both postoperative periods studied, immediate and distant poor outcome after decompression surgery and good postoperative outcome persisting one month after. **Conclusions:** The results obtained in the study and the surgical protocol proposed by the authors are in line with the current state of knowledge regarding orbital hematomas. Some of the correlations described in the literature (such as OCS and anticoagulant treatment) were not demonstrated, but this is probably due to the small study group. Maintaining the 100 min limit as a standard was possible only in early postoperative diagnoses (only 1 of the patients was operated on up to 100 min after the appearance of symptoms). In other cases, the specialized diagnosis took an average of 2785 ± 4020 min or 46 ± 67 h.

## 1. Introduction

Orbital compartment syndrome (OCS) is a rare consequence of craniofacial trauma and orbital surgery despite continuous improvements in the methods and materials used for stable osteosynthesis [1,2,3,4]. There are two major factors that play a crucial role in the pathomechanism of OCS: the closed orbital space and the increasing hydrostatic pressure exerted by the blood in the retrobulbar region on the optic nerve and retinal cells [5]. In the course of OCS, a progressive loss of visual acuity is observed that, if untreated, leads to total blindness [6,7]. It has been shown that the first irreversible changes in the retina are observed as early as 60–90 min into the course of OCS [8].

It is noteworthy that not every hematoma causes Orbital Compartment Syndrome, and in fact most hematomas observed do not cause this pathology and resolve without treatment or consequence [5]. The vast majority of subperiosteal bleeds diagnosed on CT performed for orbital fracture do not require intervention and do not progress to OCS. In fact, subperiosteal or retrobular bleeding is common in patients after trauma and affects up to 30% of patients. They do not cause retinal ischemia or optic nerve neuropathy. Such symptoms occur in 0.5–1% of trauma patients [9,10].

Diagnosis and treatment of OCS should be based on clinical symptoms (decreased visual acuity, periorbital pain, proptosis, eyelid edema and restriction of extra-ocular motility), as observing a hematoma on CT is not a predictor of acute or future orbital compartment syndrome. However, if symptoms of OCS are found, decompression should not be delayed [5].

The purpose of this study was to retrospectively evaluate the results of treatment of orbital hematomas and attempt to standardize the management protocol based on the literature and the authors’ own experience.

## 2. Materials and Methods

The study included 18 patients treated at the Department of Maxillofacial Surgery of Medical University of Lodz Poland and Clinical Department of Maxillofacial Surgery of Medical University of Poznan Poland between 2009 and 2022 due to OCS. All orbital injuries in Lodz and in Poznan were consulted ophthalmologically. Cases of extraocular hematoma were diagnosed using CT of the head region, read by both the radiologist and members of this research team. Raw evaluation was carried out in RadiAnt (Medixant, Poznan, Poland, www.radiantviewer.com, accessed on 9 April 2024)

Hematoma was measured according to the following protocol: CT images in Digital Imaging and Communications in Medicine format were segmented and transformed into a 1-bit three-dimensional model based on individual histogram analysis according to the Baillard and Barillot protocol [11] using Mimics 17.0 software (Materialise, Leuven, Belgium, www.materialise.com, accessed on 9 April 2024). Values corresponding to a hematoma were selected and measured using the stability function in freeware Meshmixer software (Autodesk, San Rafael, CA, USA, www.autodesk.com, accessed date 9 April 2024).

The following factors were considered from the database of electronic patient records: systemic diseases, cause, location of the hematoma, presence and number of fractures, visual impairments (diplopia, decreased visual acuity, only light perception retained, blindness) and pupillary reaction (normal, prolonged pupil contraction, no pupil contraction) on admission to the hospital.

In addition, OCS cases were evaluated for the type of intervention and the time that elapsed between admission to the hospital and surgery.

The presence of visual disturbances on the first day after surgery and one month after were also considered.

Statistical analysis was performed in Statgraphics Centurion 18 (Statgraphics Technologies Inc. The Plains, VA, USA). A *p*-value < 0.05 was taken as statistical significance, corresponding to a 95% confidence level.

## 3. Results

The patients’ ages ranged from 13 to 90 years (median 53.4 years), including three women and fifteen men.

**Figure 1:**
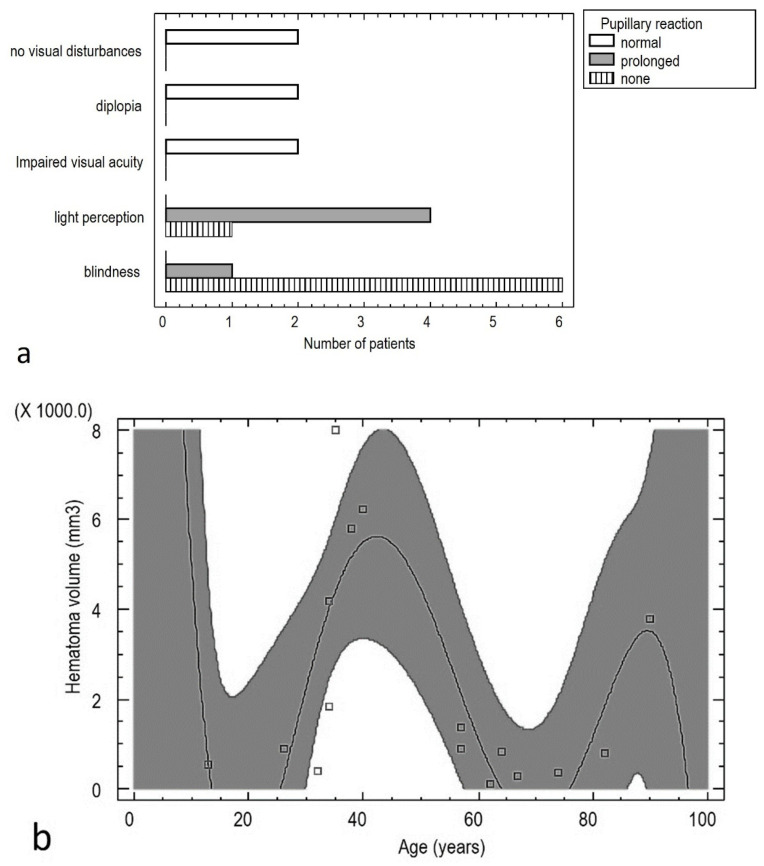
Relationship between pupil response to light and visual impairment (**a**), and the relationship between the volume of the retrobulbar hematoma and the age of the patients (**b**).

Statistically significant correlations were obtained between number of systemic diseases and patients’ age (Pearson correlation = 0.68; *p* < 0.01) and the degree of visual impairment and pupillary reaction [*p* < 0.001]. A statistically significant relationship was also found between hematoma volume and patient age [*p* < 0.05]. The relationship can be described by the formula below (Figure 1):

Hematoma volume (mm^3^) = 47,586.4 − 7243.61 × Age (years)^2^ + 372.43 × Age (years)^2^ − 8.2075 × Age (years)^2^ + 0.080902 × Age (years)^3^ − 0.000293584 × Age (years)^5^

**Figure 2:**
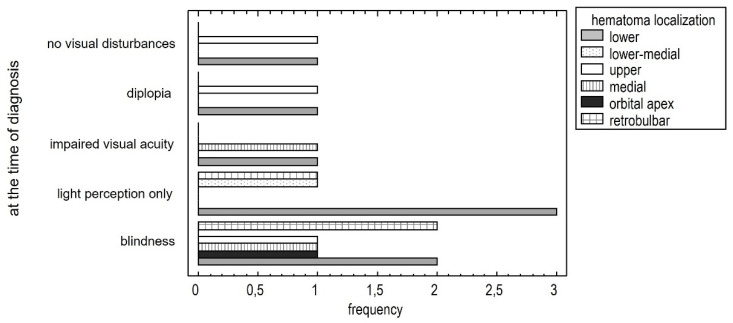
Relationship between severe visual impairment and hematoma localization.

As the symptoms worsen, new locations appear. Initially, symptoms occur in the medial area, then the inferior lower area and orbital cone. The occurrence of increasingly severe symptoms correlates with the location of the hematoma [*p* < 0.05]. (Figure 2).

**Figure 3:**
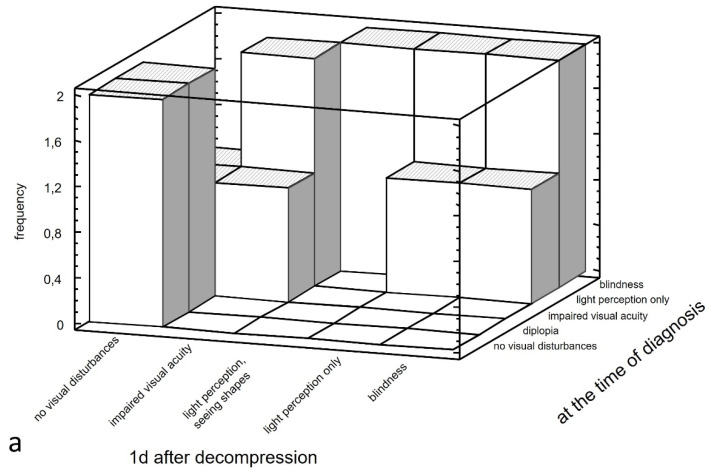

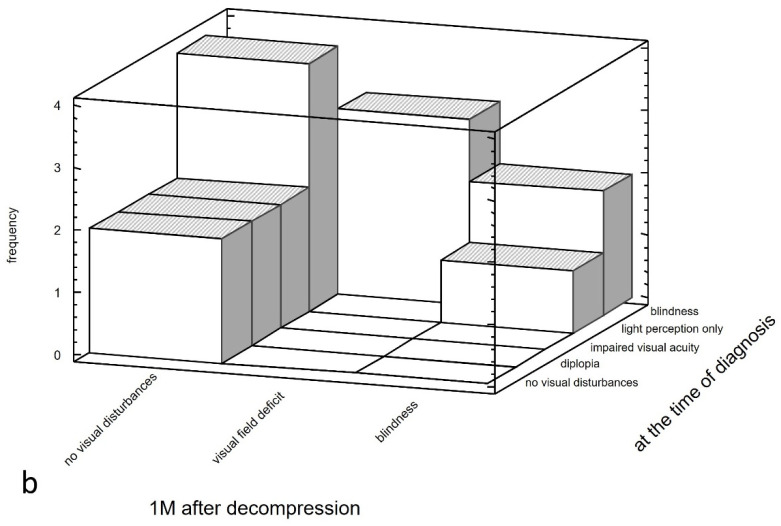
Relationship between severe visual impairment at the time of diagnosis (blindness, light perception only, impaired visual acuity, diplopia) and on the first day after surgery (blindness, light perception only, light perception, seeing shades, impaired visual acuity) (**a**) and between severe visual impairment at the time of diagnosis (blindness, light perception only, impaired visual acuity, diplopia) and one month after surgery (blindness, visual field deficit) (**b**).

Positive statistically significant correlations were also found between severe symptoms at the time of diagnosis and poor treatment results on the first day after hematoma decompression [*p* < 0.01] and one month after hematoma decompression [*p* < 0.05]. Simultaneously, the good functional visual outcome on the first day after hematoma decompression is maintained one month after surgery [*p* < 0.001]. (Figure 3).

## 4. Discussion

The study results align with current medical knowledge on OCS. However, not all known risk factors for ocular complications were considered in the study, such as visual recovery in less than 30 h and posterior ocular angle less than 120 degrees [7].

Hematomas appear in new locations as symptoms worsen, but not randomly. Initially, OCS symptoms manifest when the hematoma is located medially in the orbit, where the two ethmoid arteries are. Subsequently, symptoms appear inferiorly, where the suborbital artery is, and finally in the orbital cone.

Practice-relevant prognostic factors for OCS are important. Our study confirmed some of the relationships known from the literature, such as the size of the hematoma, the patient’s age, the degree of visual disturbance and the weakening of pupillary constriction [7]. This last factor in particular is unfavorable prognostically. In cases of surgical intervention, statistically significant correlations were found between severe initial symptoms and poor postoperative outcomes in both the immediate and distant postoperative periods. Additionally, good postoperative outcomes were observed one month after surgery. These findings suggest that both positive and poor immediate decompression outcomes may be functionally permanent.

The present study did not include any patients who experienced bleeding as a result of antithrombotic or anticoagulant treatment, which is a known cause of OCS [12,13,14,15]. Although we did not find any statistically significant relationships between coagulation disorders and OCS, this may be due to the small size of our patient group.

The statistically significant relationship found represents three peaks of increased hematoma volume. <15, 40–50 AND >80 years of age. Typically, age >50 years is reported as a risk factor for hematoma within the orbital tissues. The association described by the authors including also the <15 years group is probably due to the small study group and requires further research in this aspect [16].

It is also important to consider the time between OCS diagnosis and decompression. In cases of large hematomas, intervention may be delayed due to the size of the injury and the limited chance of restoring vision in patients with torn eyeballs. Only one of the patients included in the study underwent surgery within 100 min of symptom onset. On average, surgical treatment was initiated 46 ± 67 h after the onset of OCS symptoms. The authors observed repeat imaging studies ordered to assess hematoma growth, which is known to have no relevance in OCS [5,17,18]. Moreover, in the realities of Polish health care, the initial symptoms of OCS were mainly detected in general hospitals where physicians could not perform decompression and opted to transfer patients to a facility with a Maxillofacial Surgery Unit. This highlights a significant flaw in the public health care system. The authors suggest that every physician working in the emergency department, irrespective of their specialization, should be capable of performing a lateral canthotomy as a first-line treatment [5,7,9,18,19].

The authors’ centers use the following protocol, which we recommend to readers when OCS symptoms are found on clinical examination qualifying the patient for surgical intervention:

1. Early hemostatic intervention (if possible).
2. Drainage of the site of the hematoma.
3. Coagulation of bleeding sites (usually venous or diffuse).
4. Wound irrigation with tranexamic acid (Exacyl, Herajid, Ugurol).
5. Possibly apply adhesive dressings to the bleeding surface with preserved bone support (e.g., lower wall), such as a Haemopatch or Tachoseal.
6. Maintain passive drainage through surgical access for 1 day.
7. Postoperative recommendations include avoiding coughing or straining. Antitussives, antiemetics, and stool softeners may be necessary.
8. Body positioning after surgery should involve elevating the head at a 45-degree angle.
9. Cold can be applied to the eyelids by pouring icy saline solution over the gauze copper dressing.
10. Pharmacotherapy for this condition may include corticosteroids such as Methylprednisolone (500–1000 mg/dz) or possibly Dexamethasone (24 mg/day), as well as Diuretics, Mannitol and Carbonic Anhydrase Inhibitors like Acetazolamide.
11. Normalization of blood pressure and coagulopathy.
12. Patient cooperation (reporting any alarming symptoms such as an increase in pain, exophthalmos, or deterioration of visual acuity).
13. The patient should be observed hourly for several hours after the procedure
14. The drain should be removed and the wound sutured.

## 5. Conclusions

The results of the study and the proposed surgical management scheme are in line with current knowledge of orbital hematomas, and can help with effective treatment at specialized medical centers. Nevertheless, it is important to remember that there are two crucial elements in the treatment of OCS: diagnosis based on clinical symptoms (mainly progressive loss of vision) and the time between diagnosis and surgical intervention, which must not exceed 60–90 min.

## Data Availability

Data are available in a publicly accessible repository Bielecki-Kowalski, Bartosz; Kozakiewicz, Marcin (2024). Stat1.docx. figshare. Dataset. https://doi.org/10.6084/m9.figshare.25805227.v1.
